# Severe Hypophosphatemia Following Ferric Carboxymaltose in a Patient With Inflammatory Bowel Disease

**DOI:** 10.7759/cureus.20452

**Published:** 2021-12-16

**Authors:** Abuobeida Ali, Ali Elmdaah, Ahmed Mohammed Mustafa, Aravind Sunderavel Kumaravel Kanagavelu, Nader Mohamed, Saber Sayed

**Affiliations:** 1 Gastroenterology, Peterborough City Hospital, Peterborough, GBR; 2 Internal Medicine, Peterborough City Hospital, Peterborough, GBR

**Keywords:** iron deficiency anemia, ulcerative colitis., inflammatory bowel disease, ferric carboxymaltose, hypophosphatemia

## Abstract

Inflammatory bowel disease is a chronic inflammatory condition that affects the large and small bowel, which includes Crohn’s disease (CD) and ulcerative colitis (UC). Iron deficiency anemia (IDA) is one of the most common complications in people with inflammatory bowel disease. The treatment of choice is intravenous iron infusion. There is a lack of awareness of side effects of intravenous iron (Ferinject) such as hypophosphatemia, which can prolong hospital admission. We present the case of a patient with iron deficiency anemia and vitamin D deficiency who developed severe hypophosphatemia after intravenous injection of ferric carboxymaltose (Ferinject). In this case presentation, our aim is to increase the awareness of prescribers about the risk of developing low phosphate levels after Ferinject and the need to monitor serum phosphate levels.

## Introduction

According to the World Health Organization (WHO), anemia is defined as hemoglobin levels of <13.0 g/dL in adult males, <12.0 g/dL in non-pregnant adult women, and <11 g/dL in pregnant women [1]. Recent randomized controlled trials showed that intravenous ferric carboxymaltose leads to a greater resolution of iron deficiency anemia (IDA) compared with those who received intravenous iron sucrose or oral ferric maltol. Taking a necessary precautionary measurement of vitamin D, parathyroid hormone (PTH), and phosphate levels could decrease the incidence of occurrence of hypophosphatemia. Phosphate is regulated by parathyroid hormone (PTH) and fibroblast growth factor 23 (FGF23) by controlling its absorption, renal excretion, and metabolism. Approximately 70% of phosphates are absorbed in the small intestine in the presence of 1,25 (OH)_2_ vitamin D3 (calcitriol) through sodium phosphate transporter type 2b [2]. Some studies suggested that iron infusion increases FGF23 by inhibiting its degradation and, therefore, this may lead to increased renal excretion of phosphate [3]. As most patients will require prolonged admission for intravenous phosphate replacement, this has led to an increased need for the use of anti-FGF23 antibody (KRN23). The first case of IV iron-induced hypophosphatemia was reported in New Zealand in 2009, and despite this, there is still a lack of awareness of the serious side effects [4]. Here, we present a case of a patient with iron deficiency anemia and vitamin D deficiency who developed severe hypophosphatemia after intravenous injection of ferric carboxymaltose (Ferinject).

## Case presentation

A 40-year-old man of Asian origin was admitted to Peterborough City Hospital with acute onset of bloody diarrhea for two weeks, associated with abdominal pain on the left side. He had a history of asthma and ulcerative colitis diagnosed in January 2016. Since the diagnosis of ulcerative colitis, he had four flares, three of them treated with infliximab in May 2016, October 2017, and recently in August 2021. Before admission, azathioprine had been intermittently started and stopped based on disease status. No significant family history of irritable bowel disease was reported. He had a normal development as a child, took inhalers for asthma, was a previous smoker, and denied alcohol consumption.

On admission, he was tachycardic with a heart rate of 108 beats per minute (bpm) and his blood pressure was 130/81 mmHg. Physical examination revealed that his height was 168 cm, weight was 65 kg, and BMI was 23. He had wheeze in the middle and lower zones of his right lung, normal cardiac auscultation findings, and the abdomen was soft but tender on the left side. His general appearance did not indicate any bone or facial deformity.

Blood tests at admission showed hemoglobin was 100 g/L (130-180), mean corpuscular volume (MCV) was 79.3 fL (80-100), ferritin was 25 ug/L (30-400), and iron <5.0 umol/L (14-29). His B12, folate, and thyroid-stimulating hormone (TSH) were within the normal range. His adjusted calcium was 2.35 mmol/L (2.20-2.60) and fecal calprotectin (>60000) was very high. Stool cultures were negative for routine polymerase chain reaction (PCR) and *Clostridium difficile* toxins. Flexible sigmoidoscopy showed that the colonic mucosa was edematous with superficial ulcers and without a vascular pattern. The abdominal computerized tomography scan showed findings consistent with pancolitis with no evidence of toxic megacolon or bowel infarction.

Intravenous hydrocortisone 100 mg four times per day, intravenous fluids, and thromboprophylaxis with dalteparin were started according to trust guidelines. Based on Travis score, he was assessed on day three which showed he responded to steroids. He was seen by the surgical team during the first week of hospitalization and there was no concern of toxic megacolon. He was also seen by inflammatory bowel disease (IBD) nurse specialist who provided the patient with some consultation on infliximab including increased risk of infection, lymphoma, skin cancer, live vaccines, non-live vaccine (influenza, pneumonia, and coronavirus disease {COVID} vaccine), blood monitoring for infusions, treatment regimen, and safety netting. The first dose (5 mg/kg) of infliximab was administered without any side effects. IBD nurses arranged an outpatient appointment for infliximab administration.

He was also treated for IDA with parenteral ferric carboxymaltose (Ferinject) at a dose of 1000 mg as per weight and hemoglobin level. He had no previous iron transfusions. The phosphate level prior to iron infusion was normal at 1.27 mmol/L (0.80-1.50). The next day, his phosphate level dropped to 0.40 (0.80-1.50) [5,6]. He also began to experience fatigability in the lower extremities on day three after intravenous iron infusion, which is one of the clinical signs of severe hypophosphatemia [7]. With the diagnosis of severe hypophosphatemia, the cause was investigated and the parathormone level was 4.3 pmol/L (1.6-6.9). Vitamin D was inadequate at 32 nmol/L (normal level>50 nmol/L). He was started on vitamin D replacement with 20,000 IU alternative days for a total of 45 doses and then switched to oral vitamin D maintenance dose at 800 IU according to hospital guidelines. He was arranged for follow-up to measure serum calcium levels in four to six weeks. We also measured the concentration of phosphate and creatinine in a fasting spot urine sample to evaluate if there was any renal loss of phosphate (urine phosphate: 14.10 mmol/L).

He was started on oral and intravenous phosphate replacement. His phosphate level remained low despite adequate replacement on the ward for 19 days prior to normalization. Reviewed several times by the nutrition and dietetics team with the impression of not meeting estimated nutritional requirements due to non-compliance with nutritional supplements and elevated nutritional requirements. Recommendation of the nutrition and dietetics team at that time, the patient should follow a lower fiber diet while in acute flare-up. Advised to avoid eating salad, and to eat only canned and cooked food. The patient received a low-fiber advice sheet. Furthermore, the nutrition and dietetic team advised to stop Fortijuce (nutritional supplement) and asked the patient to try Fortisip Compact (nutritional supplement) three times a day between meals instead, and also to closely monitor with food and stool charts.

## Discussion

Hypophosphatemia is defined as a low blood phosphate level of less than 0.8 mmol/L. Common causes of hypophosphatemia are decreased oral intake and redistribution of body phosphate, such as refeeding syndrome, respiratory alkalosis, and as a complication of diabetic ketoacidosis (DKA) management. There are other etiological factors that are related to decreased intestinal absorption and renal loss caused by hyperthyroidism or renal tubular acidosis.

Hypophosphatemia has previously been reported to occur after intravenous iron infusion (specifically ferric carboxymaltose). However, the severity of symptoms has only been reported more recently. The severity of symptoms includes central nervous system impairment in terms of confusion and can also lead to proximal muscle weakness and rhabdomyolysis. A study has shown a correlation between the severity of hypophosphatemia and the dose of administration, and around 51% of patients who received infusions of Ferinject (ferric carboxymaltose) developed low phosphate levels [6]. In our case, the main symptom developed after iron infusion was fatigue. We have noticed the lack of awareness of these potential side effects like hypophosphatemia despite multiple case reports in the literature review [8].

Preexisting problems in phosphate homeostasis, including low levels of vitamin D, calcium, and phosphate, have also been reported to increase the potential side effects of ferric carboxymaltose infusion and develop symptomatic hypophosphatemia. Our patient had vitamin D deficiency with normal calcium level and parathyroid hormone levels [9].

The literature has explained the connection of excess FGF23 and the cause of low phosphate through renal phosphate wasting [10]. In this case, we measured urinary phosphate, tubular maximum reabsorption of phosphate/glomerular filtration rate (TmP/GFR), and both were normal. However, we have found that our patient had a low level of vitamin D and this could have also increased the risk of developing symptomatic hypophosphatemia after iron infusion replacement.

According to the hospital protocol, the maximum dose of IV phosphate that can be administered with the need of cardiac monitored bed is 90 mmol over 12 hours of infusion. Despite daily intravenous and oral replacement of phosphate, this took almost 19 days before serum phosphate normalized in the patient and stabilized.

## Conclusions

Hypophosphatemia is identified as a complication after infusion of ferric carboxymaltose in people with iron deficiency anemia. This complication is not recognized by the prescriber and can lead to prolonged hospital admission. Identifying people who are at risk, such as vitamin D deficiency, might decrease the risk of developing such complications. A recent update of the British Society of Gastroenterology (BSG) guidelines regarding IDA treatment provides the percentage of iron formulations that carry a high risk of developing hypophosphatemia.
